# Real-world behavioral dataset from two fully remote smartphone-based randomized clinical trials for depression

**DOI:** 10.1038/s41597-022-01633-7

**Published:** 2022-08-27

**Authors:** Abhishek Pratap, Ava Homiar, Luke Waninger, Calvin Herd, Christine Suver, Joshua Volponi, Joaquin A. Anguera, Pat Areán

**Affiliations:** 1 Krembil Centre for Neuroinformatics, Centre for Addiction and Mental Health, Toronto, ON Canada; 2 Department of Psychiatry, University of Toronto, Toronto, ON Canada; 3 Vector Institute for Artificial Intelligence, Toronto, ON Canada; 4 Institute of Psychiatry, Psychology & Neuroscience, King’s College London, London, UK; 5 Department of Biomedical Informatics and Medical Education, University of Washington, Seattle, WA USA; 6 School of Interdisciplinary Science, McMaster University, Hamilton, ON Canada; 7 Sage Bionetworks, Seattle, WA USA; 8 Department of Neurology, University of California San Francisco, San Francisco, WA USA; 9 Department of Psychiatry & Behavioral Sciences, University of Washington, Seattle, WA USA

**Keywords:** Health care, Research data

## Abstract

Most people with mental health disorders cannot receive timely and evidence-based care despite billions of dollars spent by healthcare systems. Researchers have been exploring using digital health technologies to measure behavior in real-world settings with mixed results. There is a need to create accessible and computable digital mental health datasets to advance inclusive and transparently validated research for creating robust real-world digital biomarkers of mental health. Here we share and describe one of the largest and most diverse real-world behavior datasets from over two thousand individuals across the US. The data were generated as part of the two NIMH-funded randomized clinical trials conducted to assess the effectiveness of delivering mental health care continuously remotely. The longitudinal dataset consists of self-assessment of mood, depression, anxiety, and passively gathered phone-based behavioral data streams in real-world settings. This dataset will provide a timely and long-term data resource to evaluate analytical approaches for developing digital behavioral markers and understand the effectiveness of mental health care delivered continuously and remotely.

## Abstract (summary)

**Table:** 

Measurement(s)	depression • real-world behavior • anxiety • Depressed Mood • physical mobility • daily smartphone usage
Technology Type(s)	Smartphone Application • GPS navigation system
Factor Type(s)	depression level, socioeconomic indicators, demographics
Sample Characteristic - Organism	Homo sapiens
Sample Characteristic - Environment	real world setting
Sample Characteristic - Location	contiguous United States of America

## Background & Summary

Although effective treatments exist, depression is one of the leading causes of disability worldwide^1^. A challenge in eradicating this burdensome and costly illness is the poor access to timely diagnosis and treatment. Individuals with depression often lack adequate and timely care, and even when care is available, it is often not evidence-based, and outcomes are not measured consistently^1–3^. In addition, there are known barriers such as time commitment, regular follow-ups in behavioral therapy^4^, and limited availability of highly trained professionals, especially in rural and lower-income areas^5^. Some populations also do not access care because of the stigma associated with mental health problems^6^. In particular, there are known socio-technical challenges in recruiting underserved communities^7^ such as the Hispanic/Latino population, in traditional research studies^8,9^ in the US. Asian, Hispanic, and Black people are less likely to receive access to and utilize mental healthcare services than their White counterparts^10–12^. As a result, our understanding of optimal support and treatment options for underserved and minoritized populations remains limited.

In response to such challenges, there is broad interest in using digital health technology to provide individuals with novel preventative and therapeutic solutions in a timely and scalable manner compared to traditional mental health services^13^. Current applications of digital technology in mental health include the remote deployment^14^ of guided interventions (e.g., cognitive behavioral therapy^15^). Due to heterogeneity in diagnoses of mental illnesses, researchers are beginning to explore the use of smartphones and wearables^16^ in understanding personalized day-to-day factors and their severity impacting individuals’ mental health. Multimodal data gathered from connected devices can help understand individualized real-world behavior, aiding the development of digital phenotypes^17^. Once validated, these digital signatures can ultimately help track individual physiological symptoms that can be used to tailor and augment the treatment of mental illnesses^18^ as well as guide the development of novel treatments for mental health disorders.

In the last ten years, technology developers and researchers have begun to explore the potential of smartphone apps as an economical and scalable means for assessing and delivering mental health care. Over 10,000 smartphone apps for mental health and well-being are available on the App and Play stores. However, there is little information about their utilization, efficacy, and effectiveness^19^. When assessing the quality and efficacy of smartphone-based interventions as a supplement to treatments or compared to other therapeutic interventions, there have been varying results across treatment groups^20^. There have also been notable concerns regarding the use of mobile apps, potentially excluding groups who may not have access to technology due to cost, digital literacy, location, or availability^21^. Many individuals also express concerns over potential privacy and consent issues regarding access to personal mental health-related data^22^ and apps storing sensitive information such as account information and demographics, which may discourage their use^23^. Essential questions regarding the utility of digital mental health apps are (1) who downloads these apps, (2) do people who use these apps use them as designed, (3) are they effective in collecting valid and long-term behavioral data, (4) for whom are they effective and for how long, and (5) are these tools easy to access and as effective in under-represented minorities?

The limited availability of real-world behavioral datasets curated using FAIR^24^ data sharing and management principles is a critical bottleneck that hampers digital mental health research advancement. A recent systematic review^25^ of real-world behavior sensing studies highlighted substantial challenges such as small sample sizes and inconsistent reporting of analytical results that affect the reproducibility of findings. And as a result, it impacts the development of robust and transparently validated digital biomarkers of mental health using real-world data (RWD). Irreproducibility of research findings remains the top challenge stymieing the real-world application of biomedical research. Recent meta-studies have shown that up to 50% of reviewed studies were not reproducible, costing taxpayers billions of dollars every year^26,27^. NIH has recently instituted a mandate requiring researchers to make their research data accessible for all NIH-funded research starting January 2023^28^.

We describe and share one of the most extensive longitudinal real-world behavioral datasets to enable a broader ecosystem of digital health datasets accessible to the biomedical research community and curated using FAIR principles^24^. We believe this dataset will provide a significant long-term value for the researchers interested in digital mental health across various use cases, such as (1) cross-study data integration and comparisons of findings, (2) benchmarking analytical approaches for predicting functional and behavioral outcomes, (3) specific app use signatures and their relationship to outcomes, (4) modeling individualized behavioral trajectories over time, (5) the impact of being in traditional treatment while using the app on outcomes, (6) discovery depression subtypes based on baseline characteristics and how they relate to: i) adherence with care, ii) changes in mood and sleep patterns, and iii) stability of outcomes.

## Methods

### Brighten V1 and V2 studies

The two randomized clinical trials, Brighten (Bridging Research Innovations for Greater Health in Technology, Emotion, and Neuroscience) V1 study^29^, (henceforth V1) and Brighten V2^7^, (henceforth V2), have been used to investigate: 1) feasibility of conducting fully remote decentralized clinical trials^7,29^, 2) the relative effectiveness of digital health apps in improving mood^30^, 3) utility of real-world behavioral data collected passively from smartphones in predicting changes in mood over time at the population and individual level^31^ and 4) factors impacting participant recruitment and retention in decentralized health research^32^. Both studies deployed three app-based remote interventions and gathered real-world data (active: remote surveys and passive: smartphone usage) related to depression shared and described here.

**Fig. 1:**
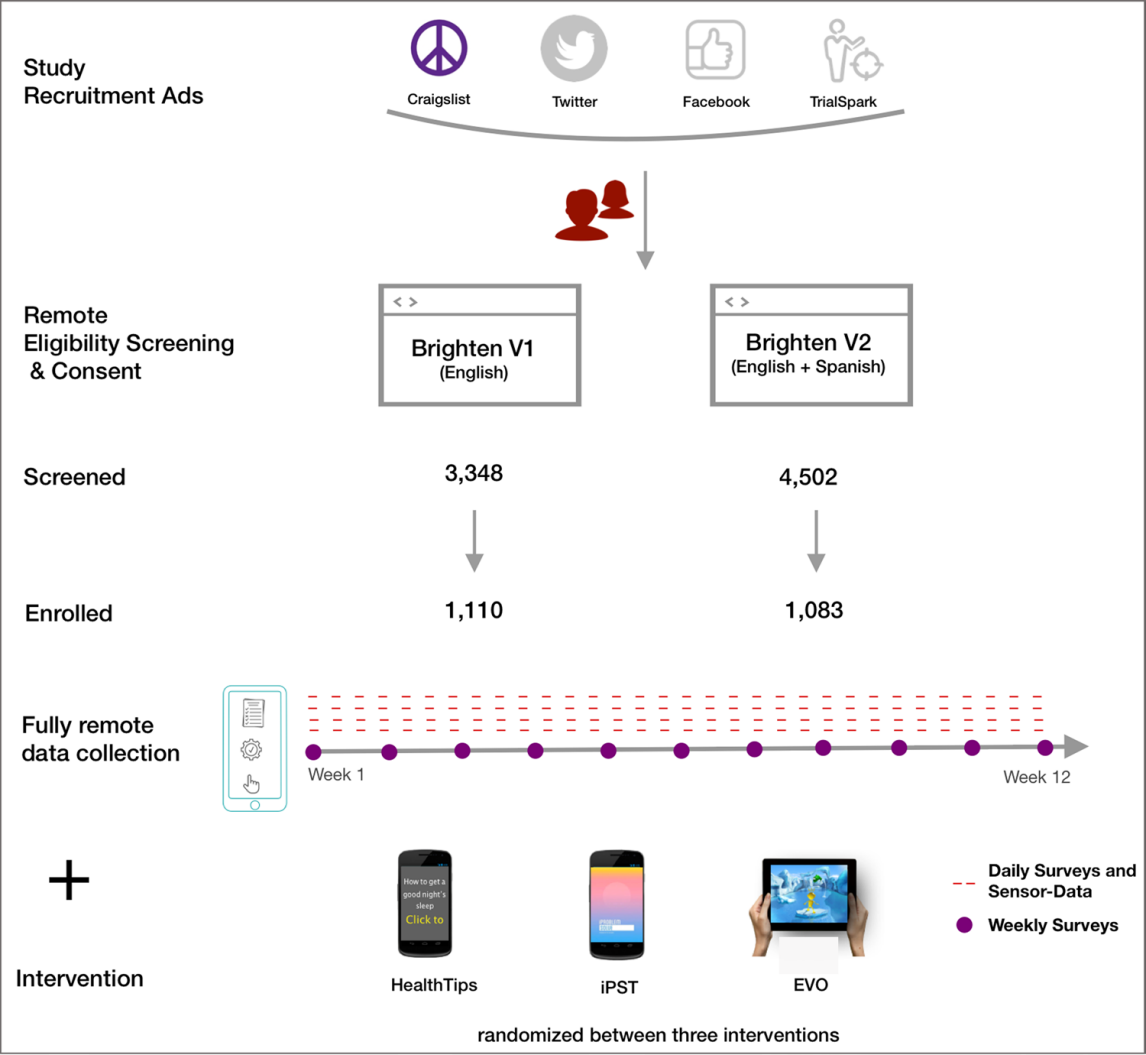
Overall schematic showing participant onboarding process from online recruitment and prospective data collection using smartphones to random allocation to one of the study interventions.

**Fig. 2:**
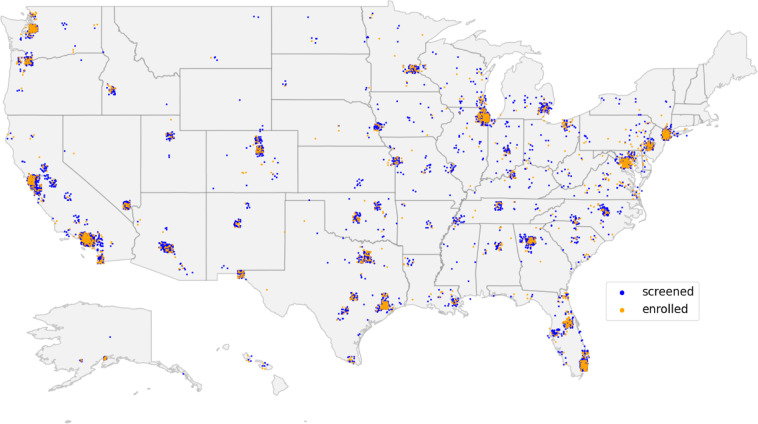
US map showing the location of participants* who were screened (blue) and enrolled (yellow) in the two Brighten studies. *Based on participants who shared at least the first three numbers of their zipcode.

Overall, 7,850 participants from across the US were screened, with 2,193 participants meeting the eligibility criteria and consenting to enroll in the two studies (Figs. 1, 2; Table 1). The individual-level engagement data analysis showed significant variation in participant retention linked to demographic and socioeconomic factors further described within the two studies^7,32^. Study participants’ average depression severity (assessed using PHQ-9 survey^33^) at onboarding indicated moderate depression symptoms (V1:14 ± 5.1, V2:14.3 ± 5.4). A notable proportion of the cohort indicated suicidal ideation (non-zero response to 9^th^ item of PHQ-9) at onboarding (V1:29.4%, V2:35.6%).

**Table 1:** Summary of all surveys and passive data contributed by participants enrolled in the two studies along with data citation.

Administration Frequency	Activity Type	Content	Unique Participants	Total Records	Data Citation
Once	Baseline Characteristics	Age, Gender, State, Zip Code, Income, Race Education, Study Arm, Device	2193	2193	10.7303/syn27082597^47^
	Baseline PHQ-9	A clinically validated screener of depression was used to screen participants for eligibility of joining the study	1919	1919	10.7303/syn27082811^48^
	GAD-7	A clinically validated screener of generalized anxiety disorder	820	820	10.7303/syn17022655^49^
	AUDIT-C	A three-item alcohol screener	832	835	10.7303/syn17021280^50^
	IMPACT Mania and Psychosis Screening	A 5-question self-assessment survey used to screen for issues of mania and/or psychosis collected during the onboarding week	818	825	10.7303/syn27051276^51^
Weekly	PHQ-9	Same as baseline PHQ-9 survey administered weekly for the first four weeks and then every 2 weeks	934	4875	10.7303/syn27202355^52^
	SDS	A clinically validated tool to assess functional impairment administered weekly for the first four weeks and then every 2 weeks	1016	4761	10.7303/syn17022658^53^
	Patients Global Impression of Change Scale	Reflects a patient’s belief about the efficacy of treatment	915	2856	10.7303/syn17023313^54^
	Sleep	A three-question survey to assess the participant’s personal sleep characteristics.	948	2319	10.7303/syn17022659^55^
	Mental Health Services Used	Five-question survey to assess the use of mental health services	947	2324	10.7303/syn17022660^56^
	Other health-related apps used	Survey to assess other health-related apps used by study participants administered during week 1,4,8,12	818	2022	10.7303/syn17025058^57^
	Study App Satisfaction	A four-question survey to probe participants satisfaction with the assigned intervention app deployed at week 4,8 and 12	514	1175	10.7303/syn17025202^58^
Daily	PHQ-2	This survey contains the first two questions of PHQ-9 focused on assessing mood and anhedonia and was administered daily during the 12 weeks of the study period	1073	47,976	10.7303/syn17020855^59^
	Passive data	Tracking sensor-based data such as phone usage and GPS. For Brighten-v2 raw GPS data was collected	900	60,470	See Table 3

There were significant differences in race/ethnicity between the two study cohorts (Hispanic/Latino V1:13%, V2:38%) primarily driven by underlying differences in recruitment approaches between studies. Using targeted recruitment approaches (further described here^7^), we enriched the V2 study cohort for Hispanics/Latinos to help investigate the feasibility of using digital mental health tools in this target population. See Table 2 for further details and a descriptive summary of the overall cohort stratified by V1 and V2 studies.

**Table 2:** Sociodemographic summary of enrolled participants in Brighten V1 and V2 studies.

	Overall	Brighten-V1	Brighten-V2
***Screened(N)***	7850	3348	4502
***Enrolled (N)***	2193	1110	1083
**Gender (%)**			
Female	1638 (74.7)	870 (78.4)	768 (70.9)
Male	547 (24.9)	235 (21.2)	312 (28.8)
**Age Group (%)**			
18–30	1039 (48.4)	583 (53.6)	456 (43.1)
31–40	563 (26.2)	243 (22.4)	320 (30.2)
41–50	350 (16.3)	151 (13.9)	199 (18.8)
51–60	147 (6.9)	84 (7.7)	63 (6.0)
61–70	38 (1.8)	20 (1.8)	18 (1.7)
71+	8 (0.4)	6 (0.6)	2 (0.2)
**Device (%)**			
iPhone	1489 (67.9)	573 (51.6)	916 (84.6)
Android	551 (25.1)	384 (34.6)	167 (15.4)
**Race/Ethnicity (%)**			
Non-Hispanic White	1144 (52.2)	646 (58.2)	498 (46.0)
Hispanic/Latino	555 (25.3)	144 (13.0)	411 (38.0)
African-American/Black	222 (10.1)	138 (12.4)	84 (7.8)
Asian	149 (6.8)	94 (8.5)	55 (5.1)
More than one	70 (3.2)	70 (6.3)	0 (0.0)
American Indian/Alaskan Native / Pacific Islander	25 (1.1)	13 (1.2)	12 (1.1)
**Income Last year (%)**			
<$20,000	668 (30.5)	266 (24.0)	402 (37.1)
20,000–40,000	417 (19.0)	154 (13.9)	263 (24.3)
40,000–60,000	273 (12.4)	92 (8.3)	181 (16.7)
60,000–80,000	122 (5.6)	44 (4.0)	78 (7.2)
80,000–100,000	67 (3.1)	18 (1.6)	49 (4.5)
100,000+	130 (5.9)	31 (2.8)	99 (9.1)
**Marital status (%)**			
Single	1199 (54.7)	665 (59.9)	534 (49.3)
Married/Partner	723 (33.0)	310 (27.9)	413 (38.1)
Separated/Widowed/Divorced	255 (11.6)	130 (11.7)	125 (11.5)
**Education (%)**			
University	865 (39.4)	425 (38.3)	440 (40.6)
High School	493 (22.5)	231 (20.8)	262 (24.2)
Community College	465 (21.2)	237 (21.4)	228 (21.1)
Graduate Degree	343 (15.6)	207 (18.6)	136 (12.6)

*See Supplementary Table 1 for details on participants with missing data for one or more sociodemographic categories

### Study design

Brighten studies (V1 and V2) aimed to assess the feasibility of conducting fully remote smartphone-based randomized clinical trials to evaluate and deliver digital interventions to individuals with depression. While both trials were open to any individual meeting the eligibility criteria, Brighten V2 focused on increasing the participation of Hispanic/Latino populations. Ethical oversight and approval for both trials (V1:NCT00540865, V2:NCT01808976) were granted by the Institutional Review Board of the University of California, San Francisco. Participants in both studies were randomized to one of three intervention apps and prospectively observed for 12 weeks. The three intervention apps were: 1.) iPST - an app based on problem-solving therapy^34,35^, an evidence-based treatment for depression, 2.) Project Evo - a therapeutic video game meant to improve cognitive skills associated with depression^36^, and 3.) Health Tips - an app that provides information about strategies to improve mood.

### Participant recruitment & onboarding

Participants had to speak English or Spanish (in V2), be 18 years or older, and own either an iPhone with Wi-Fi or 3 G/4 G/LTE capabilities or an Android phone with an Apple iPad version 2.0 or newer device. In both studies, participants had to experience clinically significant symptoms of depression, as indicated by either a score of 5 or higher on PHQ-9 or a score of 2 or greater on PHQ item 10.

Participants were recruited using a combination of three different approaches - traditional methods (ads placed in local media), social media-based advertising (ads on Facebook and Craigslist), and online targeted recruitment campaigns (in partnership with Trialspark.com for the V2 study). The onboarding process involved learning about the study through the study website. A video highlighting the research study’s goals and procedures and the risks and benefits of participation was also made available.

After viewing the video and reading the online study consent, participants had to correctly answer three questions to confirm their understanding that participation was voluntary, not a substitute for treatment, and that they were randomized to treatment conditions. Each question had to be answered correctly before a participant could consent to participate, at which point they could move on to the baseline assessment and randomization. Participants’ consent to participate in the study was asked before their enrollment eligibility could be established. This was done to assess their baseline depression state (via PHQ-9 survey), which was required to meet the study eligibility criteria and could not be collected without the participant’s consent. In the consent form signed by participants, there was a clause describing that other organizations and researchers may use these data for research and quality assurance. This follows the National Institute of Mental Health (NIMH) data sharing policy (NOT-MH-19-033), designed to share data at the end of the award period or upon the research findings being published.

Once confirmed, participants were sent a link to download the study intervention app they were randomly assigned. The V2 study translated the recruitment material into Spanish for Spanish speakers. Participants were compensated for their time in both studies. Each participant received up to $75 gift vouchers for completing all assessments over the 12-week study period: US $15 after completing the initial baseline assessment and US $20 for each subsequent assessment at the 4-, 8-, and 12-week time points.

Data collection included participant self-reported data using a combination of validated survey instruments and short ecological momentary assessments. We also collected passive sensor data from smartphones related to participants’ daily activities as described below.

### Baseline assessment

All consented participants in V1 and V2 studies provided the same baseline information. The demographic survey included age, race/ethnicity, marital and employment status, income, and education. To assess the mental health status of participants at baseline, we used the following scales: The Patient Health Questionnaire (PHQ-9)^33^ for measuring depression symptoms, Sheehan disability scale (SDS)^37^ for assessing functional impairment, and Generalized anxiety disorder (GAD-7) for anxiety. The enrollment system flagged any participant indicating suicidal ideation (non-zero response on the 9th item of the PHQ-9 survey). Such participants were suggested that this study may not be the best fit for them and that they should consider reaching out to their healthcare provider. We also provided links to online resources, including a 24-hour suicide help hotline, to receive immediate help.

All participants were evaluated for a history of mania, psychosis, and alcohol consumption using a four-item mania and psychosis screening instrument^38^ and the four-item National Institute on Alcohol Abuse and Alcoholism (NIAAA) Alcohol Screening Test. We also asked study participants about smart device ownership, use of health apps, and mental health services, including the use of medications and psychotherapy. Participants were asked to rate their health on a scale of excellent to poor. All baseline assessments were collected from eligible participants before interventions were provided, and all questions were optional for participants to answer. For further descriptions of the clinical assessments and other surveys administered in the V1 and V2 studies, please visit the Brighten study data portal (www.synapse.org/brighten).

### Longitudinal data collection

The participants answered daily and weekly questions using the study app and continual passive data collection. The study app did not collect private information such as the content of text messages, emails, or voice calls.

#### Active tasks

Participants completed primary outcome assessments using PHQ-9 and SDS surveys once a week for the first four weeks and then every other week for the duration of the study (12 weeks). To assess participants’ mood and anhedonia (core symptoms of depression), we used the PHQ-2 survey administered daily in the morning. Participants also completed other secondary measures (described below) at daily, weekly, or biweekly intervals. These included sleep assessment with three questions - time taken to fall asleep, duration of sleep, and awake time during the night. Each question used a multiple-choice Likert scale of time (e.g., 0–15 minutes, 16–30 minutes, 31–60 minutes, etc.) to answer these sleep questions. Participants were automatically notified every 8 hours, for 24 hours, if they had not completed a survey within 8 hours of its original delivery. An assessment was considered missing if it was not completed within this 24-hour time frame.

#### Passive data collection

For the V1 study, we deployed an app in collaboration with Ginger.io that collected passive data. The application automatically launched the background data gathering processes once the user logged in. After the initial logon, the process was automatically launched whenever it was not already running (on phone restart or other events that terminate the process). The data collected through the device include communication metadata (e.g., time of call/SMS, call duration, SMS length) and mobile data such as activity type and distance traveled. The study app did not collect personal and identifiable communication data such as the actual text of SMS or the phone numbers for incoming and outgoing calls.

In the V2 study, we developed an in-house app named *Survetory* to help collect more fine-grained behavioral data. The passive data streams included a daily descriptive summary of phone-based communications (e.g., number of calls and texts) and intraday GPS-based locations. If location services (GPS) were available, the study app recorded the latitude and longitude every ten minutes or a movement more than 100 meters from the previously recorded spot. While the raw GPS data from participants cannot be shared broadly due to data privacy, we are sharing granular intraday level summaries of features derived from the raw GPS data. To featurize the raw GPS data, we developed an open-source pipeline, gSCAP-Geospatial Context Analysis Pipeline (https://github.com/aid4mh/gSCAP). Briefly, the gSCAP pipeline uses raw and sensitive individual-level GPS data to generate individual-level geospatial semantics that is not identifiable in the same way as GPS coordinates. Some examples of these contextual features include overall daily mobility (time and distance walking, biking, etc.), average daily weather (temperature, dew, cloud) at the participant’s location, and type of places visited in a day (coffee shop, park, shopping mall, etc.). However, the pipeline does not extract and store the location of the different places visited).

Further details about study design, participant recruitment, onboarding, and data collection are available in Brighten V1^29^ and V2^7^ papers, respectively.

### Data storage and security

The initial raw data from each study were collected and stored on HIPAA-compliant servers located in the Department of Neurology at the University of California, San Francisco. The study servers were configured to UCSF Minimum Security Standards for Electronic Information Resources standards and policies.

## Data Records

The data gathered from study participants during the 12-week observation period (as described above) can be accessed by qualified researchers^39^ via the study data portal (www.synapse.org/brighten). The study portal has detailed data descriptions for each active and passive data stream. The detailed steps for accessing the study data can be found on the study portal under the “Accessing the Brighten Study data” page and highlighted in the usage notes below.

Please refer to Table 1 for a record of surveys completed by the participants and Table 3 for passive behavioral data. Participants’ demographics and clinical characteristics at onboarding are summarized in Table 2.

**Table 3:** Summary of Passive Data contributed by participants enrolled in the two studies along with data citation.

Activity	Content	Data Citation
Passive Features Brighten v1	The passive features collected in Brighten V1 study through a propriety third-party app developed by Ginger.IO.	10.7303/syn17025500^60^
Passive Cluster Entries Brighten v2	Daily visited location categories	10.7303/syn17116695^61^
Passive Mobility Features Brighten v2	Daily aggregates of the mobility features created by gSCAP pipeline (see Methods)	10.7303/syn17114662^62^
Passive Phone Communication Features Brighten v2	Daily aggregates of the phone usage metadata such the number of incoming and outgoing calls, messages (length, sent, received), etc.	10.7303/syn17060502^63^
Passive Weather Features Brighten v2	The weather features were gathered from the Dark Sky API. For each day that GPS data existed, the API was queried for 24 hourly metrics which were aggregated into the summary statistics.	10.7303/syn17061284^64^

## Technical Validation

The two Brighten studies collected multimodal mental health and behavioral data in fully remote real-world settings of over two thousand participants and should be treated in that context. All shared data has been fully described on the study data portal as part of the data annotation and curation process. During the data cleaning and harmonization process, column names in the data files were aligned. The study portal also stores the provenance of upstream data cleaning and curation steps.

To preserve participants’ privacy and in consultation with data governance experts, we removed the self-reported zip code of study participants and the raw GPS data gathered in the V2 study. However, granular intraday GPS-derived features^40^ including the open-source pipeline gSCAP used to featurize the GPS data is freely available^41^. To optimize the parameters of the gSCAP pipeline for clustering GPS data, we took an iterative and visual approach to select parameters. Further details on pipeline parameters are available through the GitHub page (https://github.com/aid4mh/gSCAP).

Since both studies had monetary incentives for study participants to engage with the study apps over the 12 weeks, we paid particular attention to mitigating the possibility of individuals looking to take advantage of this study to acquire the research payment in several ways. First, the maximum compensation of $75 was spread over 12 weeks, reducing the motivation to “game the system.” Second, participants had to have both a valid email and phone number to successfully enroll in the study, reducing the ability to create multiple accounts. The study team also monitored for any duplicate records. Additionally, if an individual made multiple attempts to enroll using the same email address and each time did not properly answer the enrollment questions, the individual would not be enrolled. This prevents the individual from receiving access to any of the study tools or compensation. And finally, the link to download subject-specific study apps was only valid for a single user, as a unique password was required to view the download page.

### Limitations

There is active ongoing research to efficiently develop and deploy large-scale, fully remote research studies to collect health data at scale. While the early evidence shows substantial potential^42–44^ for the use of digital health technology in healthcare settings, several challenges^42,43,45^ have also emerged, such as the underrepresentation of certain populations who may lack access to the technology or the technological literacy to use the apps. These issues will need to be addressed so that the collected health-related data is representative of the target population and remains balanced over time.

The Brighten data shows similar limitations. First, potential participants with iOS and Android phones could join the study. Participants with android devices were also required to have an iOS-based tablet to use one of the interventional apps. This additional requirement impacted the cohort’s diversity, particularly in the V2 study, where significantly more participants had an iPhone. Second, both studies recruited a population not representative of the general population. For example, close to three-fourths of the enrolled participants were females and are known to be twice as likely depressed as males^46^. Third, the V2 study tried to enrich the study cohort for Hispanics/Latinos to investigate the use of digital mental health tools within that target population. This led to significant differences in the underlying race/ethnicity between the two studies and should be factored in the downstream analysis. Fourth, despite both studies providing participation incentives, we saw significant participant attrition rates, especially in the V2 study that has been further discussed in our previous work^7,32^. And finally, the passive data gathered from smartphones was impacted by underlying system-level differences in iOS and Android operating systems, along with different apps used to collect passive data in V1 and V2 studies. We suggest any downstream analysis account for such underlying differences in real-world data collection.

## Usage Notes

Brighten data have been donated by many thousands of research participants to advance our understanding of mental health. Due to the sensitive nature of the data, we implemented the following data access governance structure that respects the desire of participants to share their data while also protecting the confidentiality and privacy of research participants. Researchers interested in accessing these data need to complete the following steps:

1. Become a Synapse Certified User with a validated user profile
2. Submit a 1–3 paragraph Intended Data Use statement to be posted publicly on Synapse
3. Agree to comply with the data-specific Conditions for Use when prompted.

These include a commitment to keep the data secure and confidential, not attempt to re-identify research participants for any reason, abide by the guiding principles for responsible research use and the Synapse Awareness and Ethics Pledge(to only use the data as intended, not use the data for any commercial advertisement), and commit to reporting any misuse or data release, intentional or inadvertent to the Synapse Access Compliance Team within five business days. In addition, users must agree to publish findings in open access publications and acknowledge the research participants as data contributors and the study investigators on all publications or presentations resulting from using these data as follows: “These data were contributed by the participants of the Brighten Studies, which were funded by the National Institute of Mental Health with scientific oversight from Pat Arean, Ph.D. and Joaquin A. Anguera, Ph.D., as described in Synapse: https://www.synapse.org/brighten. See the full instructions for requesting data access on the Accessing the Brighten Data page (https://www.synapse.org/brighten)

## Supplementary information

Supplementary Information

## Data Availability

The studies used a combination of open-source and proprietary apps to collect observational data and deploy remote interventions via three apps (described above). The source code for tools used for the data collection, such as the passive data collection app (Survetory - used in the V2 study), iPST, and HealthTips (two of the three randomized interventions), is available on the study Github archive (https://github.com/aid4mh/Brighten_Studies_Archive). The gSCAP pipeline used to featurize the raw GPS data from the V2 study is available here (https://github.com/aid4mh/gSCAP). The data cleaning and curation pipeline is also available on GitHub (https://github.com/apratap/BRIGHTEN-Data-Release).
