# Supplementary material for: Real-world behavioral dataset from two fully remote smartphone-based randomized clinical trials for depression
Source: Sci Data. 2022 Aug 27;9:522. doi: 10.1038/s41597-022-01633-7 (PMC9420101; doi:10.1038/s41597-022-01633-7)
Supplement: Supplementary file 1 — Supplementary Information [file 41597_2022_1633_MOESM1_ESM.docx]

| **Supplementary Table 1. Details of participants with missing data for**  **one or more sociodemographic categories** | | | |
| --- | --- | --- | --- |
|  | **Overall** | **Brighten-V1** | **Brighten-V2** |
| ***Screened(N)*** | 7850 | 3348 | 4502 |
| ***Enrolled (N)*** | 2193 | 1110 | 1083 |
| **Gender (%)** |  |  |  |
| N/A | 8 (0.4) | 5 (0.5) | 3 (0.3) |
| **Age Group (%)** |  |  |  |
| N/A | 0 | 0 | 0 |
| **Device (%)** |  |  |  |
| N/A | 153 (7.0) | 153 (13.8) | 0 (0.0) |
| **Working (%)** |  |  |  |
| N/A | 16 (0.7) | 5 (0.5) | 11 (1.0) |
| **Race/Ethnicity (%)** |  |  |  |
| Other | 9 (0.4) | 0 (0.0) | 9 (0.8) |
| N/A | 19 (0.9) | 5 (0.5) | 14 (1.3) |
| **Income Last year (%)** |  |  |  |
| N/A | 516 (23.5) | 505 (45.5) | 11 (1.0) |
| **Marital status (%)** |  |  |  |
| N/A |  | 5 (0.5) | 11 (1.0) |
| **Education (%)** |  |  |  |
| None | 2 (0.1) | 2 (0.2) | 0 (0.0) |
| N/A | 16 (0.7) | 5 (0.5) | 11 (1.0) |
